# Magnetic Bone Tissue Engineering: Reviewing the Effects of Magnetic Stimulation on Bone Regeneration and Angiogenesis

**DOI:** 10.3390/pharmaceutics15041045

**Published:** 2023-03-23

**Authors:** Tiago P. Ribeiro, Miguel Flores, Sara Madureira, Francesca Zanotto, Fernando J. Monteiro, Marta S. Laranjeira

**Affiliations:** 1 i3S-Instituto de Investigação e Inovação em Saúde, Universidade do Porto, Rua Alfredo Allen 208, 4200-135 Porto, Portugal; 2 INEB-Instituto de Engenharia Biomédica, Universidade do Porto, Rua Alfredo Allen 208, 4200-135 Porto, Portugal; 3 FEUP-Faculdade de Engenharia, Universidade do Porto, Rua Dr. Roberto Frias, s/n, 4200-465 Porto, Portugal; 4 Porto Comprehensive Cancer Center Raquel Seruca (P.CCC), Rua Dr. António Bernardino de Almeida, 4200-072 Porto, Portugal; 5 Escola Superior de Biotecnologia, CBQF-Centro de Biotecnologia e Química Fina–Laboratório Associado, Universidade Católica Portuguesa, Rua Diogo Botelho 1327, 4169-005 Porto, Portugal; 6 Centro de Investigação Interdisciplinar em Saúde, Instituto de Ciências da Saúde, Universidade Católica Portuguesa, Rua Diogo Botelho 1327, 4169-005 Porto, Portugal; 7 Department of Information Engineering, University of Padua, Via Gradenigo 6/b, 35131 Padova, Italy

**Keywords:** angiogenesis, bone regeneration, magnetic stimulation, scaffolds

## Abstract

Bone tissue engineering emerged as a solution to treat critical bone defects, aiding in tissue regeneration and implant integration. Mainly, this field is based on the development of scaffolds and coatings that stimulate cells to proliferate and differentiate in order to create a biologically active bone substitute. In terms of materials, several polymeric and ceramic scaffolds have been developed and their properties tailored with the objective to promote bone regeneration. These scaffolds usually provide physical support for cells to adhere, while giving chemical and physical stimuli for cell proliferation and differentiation. Among the different cells that compose the bone tissue, osteoblasts, osteoclasts, stem cells, and endothelial cells are the most relevant in bone remodeling and regeneration, being the most studied in terms of scaffold–cell interactions. Besides the intrinsic properties of bone substitutes, magnetic stimulation has been recently described as an aid in bone regeneration. External magnetic stimulation induced additional physical stimulation in cells, which in combination with different scaffolds, can lead to a faster regeneration. This can be achieved by external magnetic fields alone, or by their combination with magnetic materials such as nanoparticles, biocomposites, and coatings. Thus, this review is designed to summarize the studies on magnetic stimulation for bone regeneration. While providing information regarding the effects of magnetic fields on cells involved in bone tissue, this review discusses the advances made regarding the combination of magnetic fields with magnetic nanoparticles, magnetic scaffolds, and coatings and their subsequent influence on cells to reach optimal bone regeneration. In conclusion, several research works suggest that magnetic fields may play a role in regulating the growth of blood vessels, which are critical for tissue healing and regeneration. While more research is needed to fully understand the relationship between magnetism, bone cells, and angiogenesis, these findings promise to develop new therapies and treatments for various conditions, from bone fractures to osteoporosis.

## 1. Introduction

Bone is a dynamic tissue that presents a very active remodeling capacity. However, when very large defects occur (usually >2 cm wide) due to traumatic injuries, congenital defects, surgical tumor removal, or degenerative diseases, the natural regeneration threshold is impaired. These cases require surgical intervention in order to restore bone function and healing, which can be achieved through several ways [1]. Nowadays, the gold-standard approaches include bone fixation with metallic implants, allografts, and autografts. Yet, these are not perfect solutions, since metallic implants need to be removed, allografts are associated with disease transmission, and autografts face bone availability problems and generate morbidity at the extraction site [2].

Void fillers have long been employed to solve these problems by using materials that fill the bone defects while it naturally heals. As technology evolved, scientists developed several bone scaffolds that not only fill the bone defect but also promote bone regeneration by modulating the bone and stem cells’ behavior, aiming at a faster proliferation and differentiation [3]. In terms of materials, calcium phosphates are the most explored due to their high biocompatibility, resolvability, biodegradation, and similarity with the natural bone apatite structure. However, several other materials with different properties have been used and several types combined with calcium phosphates to create multifunctional composites [4,5]. The reader is directed to another review for more detail on materials choice and development [6].

Moreover, the design of these bone substitutes also considers the structure of bones, where similarity with natural bone extracellular matrix must be kept for an optimal osteointegration and bone regeneration. In fact, bone has two distinct structures or architectures, compact and trabecular [7]. Compact (cortical) bone is located on the outer layer of long, short, and flat bones. It exhibits bone vasculature, and it is responsible for the release of minerals (when facing a deficiency) and for bone’s structural integrity, due to its high density (10% porosity) and its extensive net of collagen fibrils. On the other hand, trabecular (cancellous) bone surrounds the bone marrow in long bones and fulfils the vertebral bodies. This tissue is highly porous (50 to 90% porosity); however, it still has a great impact on the structural integrity of the tissue. Moreover, its vast surface area is essential for metabolic processes and bone turnover [8,9].

Another problem related to bone tissue engineering are implantable prostheses. These are mainly metallic structures (e.g., titanium) that need to be implanted in the bone but are inert in terms of bone remodeling and regeneration. Moreover, the integration of these constructs is rather low, a problem that is being tackled by using bioactive coatings [10]. These can be both organic and inorganic and are used to establish an interface between the prosthetic device and the natural bone as a way to improve bone attachment through regeneration and integration towards the metallic implant [11].

In bone regeneration, a critical step for optimal osteointegration of implanted devices is stimulation, which can be either chemical or physical. In terms of chemical triggers, osteoinductive growth factors such as Bone Morphogenic Proteins and Transforming Growth Factors-β were already introduced into bone scaffolds for a faster bone mineralization and cellular proliferation [12]. However, due to the low stability of these factors in scaffolds and due to being quite expensive, physical stimuli seem to be a more viable option. The most common form of physical stimuli is mechanical compression, which is easily achieved through natural locomotion. More specifically, the generated tension triggers a biophysical response that is further converted into biochemical cues, a process known as mechano-transduction [13]. The great advantage of this strategy is simplicity; however, locomotion most of the time cannot be used, and therefore, the healing rate can be very slow.

Currently, other external stimuli are being studied such as magnetic fields. These can be modulated in terms of strength and as either static or alternating fields, with studies reporting their influence on biological tissues. This review summarizes the response of bone cells and endothelial cells to magnetic fields and magnetically activated materials. Moreover, a critical discussion is provided regarding the viability of this option as a bone tissue engineering strategy.

## 2. Magnetic Fields Influence on Cell Behaviour

### 2.1. Influence of Magnetic Stimulation on Bone Regeneration

Before going into more complex systems such as bone remodeling, the influence of magnetic fields on cells is still a matter of debate. Firstly, magnetic fields can be employed as static or as alternating fields, where the first consists of a constant field with uniform strength, while the latter is described by a variation in amplitude with time. Moreover, static magnetic fields (SMFs) can be divided according to the field strength, such that intensities of lower than 5 µT generate a hypo-magnetic field, those between 5 µT and 1 mT are entitled weak SMF, moderate ones range from 1 mT to 1 T, and those above 1 T are considered high-strength SMF [14,15]. These intensities are critical in cell behavior, and generally, it has been described that weak and moderate magnetic fields are non-harmful to animals, in contrast to hypo- and high SMF [16]. On the other hand, alternating magnetic fields are generated from an electric field. In general, cell behavior is also dependent on the generated field strength; however, since these fields are produced through the application of electric fields, there is also a risk for heat production, especially in the presence of magnetic materials.

**Figure 1:**
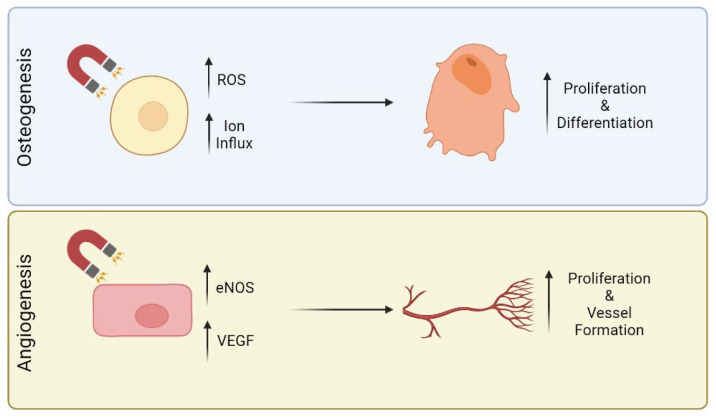
Influence of magnetic fields on osteogenesis and angiogenesis.

When it comes to cell response, SMFs have shown great potential in inducing the differentiation of progenitor cells into specialized cells. For example, Bekhite et al. showed that SMFs of 1 mT can induce cardiomyogenesis of Flk-1+ cardiac progenitor cells through calcium influx and reactive oxygen species (ROS) production. These authors explained that calcium influx was modulated by the activation of stretch-activated cation channels through the magnetic fields. Moreover, the calcium influx led to the production of nanomolar concentrations of ROS, which are key players in cellular differentiation [17]. In the specific case of bone tissue and similarly to other cells, changes in intracellular mineral ions concentration seem to be one of the triggers for differentiation, and the magnetic field strength can modulate such changes (Figure 1). To prove this, Zhang et al. (2014) exposed MC3T3-E1 cells to SMF of different strengths (500 nT, 0.2 T and 16 T) and showed that, as the field strength increased, the concentration of metallic ions such as calcium, magnesium, and iron also increased intracellularly, which in turn resulted in a higher degree of differentiation [18]. Concerning iron, this element and magnetic fields have a natural relationship, and Yang et al. (2018) described that under high SFM (16 T), iron ions accumulation inside MC3T3-E1 cells derived from an increased expression of the transferrin receptor 1 and of ferroportin 1. This effect also correlated well with increased alkaline phosphatase (ALP) activity and subsequent mineralization [14].

Besides differentiation, proliferation has also been enhanced by SMF. Lew et al. exposed dental pulp stem cells (DPSCs) to a moderate SMF (0.4 T) and suggested that the enhanced proliferation was due to a cytoskeleton reorganization and increased calcium influx [19]. Moreover, on the topic of morphological changes, Qian et al. showed that by exposing MC3T3-E1 cells to high SMF (12–16 T), the cell ultrastructure was affected, which resulted in increased cell proliferation for up to 48 h. Additionally, ALP activity increased, and collagen I and integrins were up-regulated, which is indicative of cell survival, proliferation, and differentiation [20]. In an in vivo scenario, Zhang et al. showed that moderate SMF (4 mT) could prevent bone deterioration in diabetic mice (diabetes mellitus is associated with increased fracture risk due to fast bone degradation). The study indicated that SMF induced higher osteocalcin levels, number of osteoblasts, *BMP2*, and *Runx2* gene expression, while not influencing osteoclast related biochemical factors [21]. A summary of these studies can be seen in Table 1.

### 2.2. Influence of Magnetic Stimulation on Angiogenesis

Endothelial cells play a vital role in the process of bone regeneration. They are involved in forming new blood vessels, a process known as angiogenesis, which is essential for the growth and repair of bone tissue. During bone healing, endothelial cells migrate to the site of injury, forming new blood vessels to provide the necessary oxygen and nutrients for the proliferation and differentiation of bone-forming cells. This cell type also produces various growth factors and cytokines that promote bone formation and repair, such as Vascular Endothelial Growth Factor (VEGF) and Fibroblast Growth Factor (bFGF). Moreover, they are responsible for modulating the activity of bone-resorbing cells, such as osteoclasts, to maintain an adequate balance between bone formation and bone resorption [24].

It is known that magnetic fields interact with all biological tissues; however, very few studies report their influence on endothelial cells and complex vasculature systems (Figure 1). On that topic, Martino et al. showed that weak magnetic fields positively impact the growth of endothelial cells. Those authors found that endothelial cells are sensitive to both the magnetic field’s strength and time of exposure. For 2 days at 120 µT, proliferation of HUVECs increased by 40%. In contrast, at 60 µT, with only 24 h of exposure, a 40% increase in the number of cells was observed. Such increase was justified by the augmented endothelial nitric oxide synthase (eNOS) expression. When compared with the control, the percentage of expressed eNOS was 54% against 78% with magnetic stimulation [22]. In another study, Wang et al. found that pulsed magnetic fields promoted the growth of endothelial cells in the metaphysis of long bones. The study suggested that the expansion of types H vessels may be mediated by HIF-1α signalling in these endothelial cells, when exposed to the magnetic field [23].

In normal conditions, the formation of new blood vessels is mediated by biological growth factors such as VEGF, which may be difficult in cases of existence of disease. Okano et al. studied the influence of a 120 mT SMF on endothelial cells. The results showed that the magnetic field alone stimulated cell growth and tubule formation over a period of 10 days. However, when supplemented with VEGF-A, the results were much more evident, which showed a synergistic effect between the physical stimulation and the chemical one [25]. A summary of these studies can be seen in Table 1.

## 3. Magnetic Nanoparticles Influence on Cell Behavior

### 3.1. Magnetic Nanoparticles Production Methods

Magnetic Nanoparticles (MNPs) are of great interest in biomedicine, and they are used in a wide range of applications (e.g., cell separation and guidance, magnetic contrast, and magnetic-based cancer therapies) [26,27,28]. MNPs are a class of nanomaterials, composed of metals such as iron or cobalt among others, which endow magnetic manipulation under the influence of an external magnetic field. Among all the different magnetic materials, iron-oxide-based MNPs are the most explored due to the high saturation magnetization and simple synthesis processes. More importantly, they are chemically stable, are in general biocompatible, and due to dimensionality, present a superparamagnetic behavior [29]. In terms of synthesis, there are several techniques to achieve shape-controllable, monodisperse, and stable MNPs: in fact, the synthesis of MNPs is a multistep procedure, in which physicochemical properties and stability are strictly controlled to fit several different requirements [30].

The synthesis of MNPs can be classified into three primary methodologies: physical methods (e.g., gas-phase deposition and electron beam lithography), chemical methods (e.g., co-precipitation, thermal decompositions, and hydrothermal synthesis), and biological methods (e.g., “green synthesis”). To date, the preparation of magnetic nanomaterials for biomedical applications is mainly related to chemical approaches (up to 90% of the cases) due to their simple procedure and capabilities to control the required parameters effectively. Therefore, in this section, only the chemical methods for synthesizing MNPs are presented [31].

Starting from co-precipitation, this method is perhaps the simplest approach for synthesizing MNPs [31,32]. It is described by a chemical reaction in an aqueous monophasic medium between soluble metal precursors, where a nucleus is first formed by adding a base, followed by crystal growth [33]. During this process, size, morphology, and composition of the nanoparticles can be controlled by a series of experimental parameters such as the type of precursors, the precursor ratio, the surface ligand, the reaction temperature, and the pH. For example, Bhandari et al. used a single-step co-precipitation method (basic pH by the addition of ammonium hydroxide and a working temperature of 85 °C) to synthesize functionalized MNPs with curcumin, a polyphenolic molecule. The produced particles presented sizes around 10 nm with a spherical morphology. Curcumin in this case served as an active molecule and as a capping agent [34].

The thermal decomposition method is based on the thermal decomposition of organometallic compounds, such as acetylacetonates or carbonyls, in organic solvents in the presence of surfactants, such as oleic acid and hexadecylamine. The ratio of the various precursors involved in the reaction governs the size and shape of nanostructures formed in the process. Thermal decomposition is one of the most effective methods to produce narrow size distribution MNPs, also allowing for the fine-tuning of particle mean diameter [35]. The optimal temperature required for this reaction ranges between 100 °C and 350 °C, leading to the production of MNPs with sizes between 4 and 30 nm in diameter and exhibiting a high degree of uniformity and good magnetic properties [35,36]. For example, Xie et al. produced superparamagnetic iron-oxide nanoparticles (SPIONs) by the thermal decomposition (working temperature of 260 °C) of iron (III) acetylacetonate in poly (ethylene glycol) (PEG) containing poly (ethylene imine) (PEI). The employed method produced very stable spherical nanoparticles with sizes below 11 nm. The use of functional moieties such as Tween-80, PEG, and PEI were the main contributors to the quality of the MNPs [37].

Finally, the hydrothermal method is described by a reaction between the metal precursors in various types of media, under high temperature and pressure. In more detail, the typical reaction temperature for hydrothermal synthesis is between 130 and 250 °C, while the required pressure is between 0.3 and 4 MPa. In terms of results, this technique allows for the formation of very uniform nanoparticles, with the possibility of tuning the size from a few to several hundred nanometers. Other benefits are exceptional crystallization and simple morphology control of the product, obtaining MNPs of diverse shapes, such as nanowires and nanospheres. Yet, obtaining particles with sizes below 10 nm is very challenging using this method [38]. For example, Liu et al. produced Fe_3_O_4_ nanoparticles using the hydrothermal synthesis method (working temperature of 150 °C). The nanoparticles presented good crystallinity, sizes between 10 and 20 nm, and a spherical morphology. To avoid aggregation and preserve a good dispersibility, synthesis was performed in the presence of ionic liquids that coated the surface of the nanoparticles [39].

### 3.2. Influence of Magnetic Nanoparticles on Bone Regeneration

MNPs (<100 nm at least in one dimension) present several advantages compared to their bulk counterparts, mainly due to the high surface area/volume ratio. Such dimensions allow them to be internalized by cells and to directly interact with cell receptors and other macromolecules [40]. As presented in the previous section, iron oxide (Fe_3_O_4_ or Fe_2_O_3_) nanoparticles are the most explored due to their simple production methods at the nanoscale. Moreover, they are easily functionalized and personalized and present a high magnetic response. As iron is a trace element in the human body, it can be metabolized, which contributes to the overall safety of this material [41]. In fact, regarding their effects on cells, most studies show that iron oxide nanoparticles are biocompatible and most of the time are inert to cells, unless an external magnetic field is applied.

**Figure 2:**
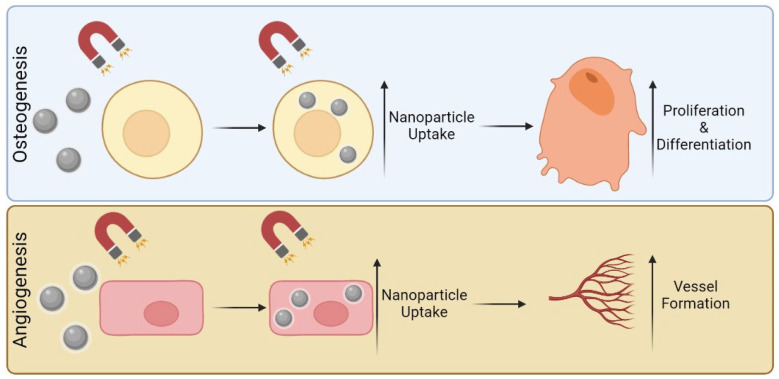
Influence of MNPs on osteogenesis and angiogenesis.

In the specific application of stem-cell magnetic targeting, iron oxide nanoparticles are internalized by stem cells, being then guided through an SMF. A study made by Silva et al. reported that stem cells remained functional after magnetic targeting (0.3–0.45 T) even though the proliferation rates decreased and cell viability was temporarily reduced. This work showed that the application of SMF in the presence of MNPs can in fact alter cell response [42]. Still on the topic of stem cells, Jiang et al. demonstrated that Fe_3_O_4_/BSA nanoparticles (200 nm) alone did not affect stem cell differentiation, nor the SMF alone (1 T). However, when combined, a higher degree of internalization of particles was achieved, which in turn significantly increased osteogenic differentiation (Figure 2). Interestingly enough, cell proliferation decreased, yet this was expected, since they were differentiating [43]. Besides stem cell therapy, MNPs can also influence pre-existing bone cells. In the case of osteoporosis, where the balance between osteoblasts and osteoclast activity is compromised, Marycz et al. found that a novel magnetic core–shell nanocomposite could re-establish the balance. In their research, it was found that the combination of Co_0.5_Mn_0.5_Fe_2_O_4_@PMMA with SMFs (0.2 T) resulted in the stimulation of integrins, which improved pre-osteoblasts activity while inhibiting osteoclasts [44]. A summary of these studies can be seen in Table 2.

### 3.3. Influence of Magnetic Nanoparticles on Angiogenesis

Regarding the use of MNPs and endothelial cells, the existing literature indicates that much remains to be explored. However, in an in vivo study by Hu et al., gelatinous sponges with SPIONs were implanted in the incisor sockets of rats. The results indicated that SPION-containing gelatine sponges showed an increase in bone density and trabecular volume/tissue volume, as well as increased new bone formation according to histological analysis, which also indicated an increase in blood vessel formation in conjunction with bone development. The SPIONs were observed to have been taken up by osteoblasts and vascular endothelial cells, leading to improved bone and blood vessel formation (Figure 2) [45].

Contrarily, Mulens-Arias et al. through an in vitro study, found that PEI-coated SPIONs negatively affected the functioning of primary HUVECs, impairing endothelial cell migration and tube formation, reducing blood vessel numbers at tumor sites. The SPIONs caused the cells to produce more reactive oxygen species, disrupting the cells’ actin cytoskeleton activity [46]. A summary of these studies can be seen in Table 2.

## 4. Magnetic Scaffolds in Bone Tissue Engineering

### 4.1. Production Methods of Magnetic Bone Scaffolds

Magnetic scaffolds found application in bone tissue engineering as a guiding structure intended to promote bone tissue formation, bone repair, and regeneration. These scaffolds should ideally have a physicochemical composition and mechanical properties close to that of native bone.

Magnetic-responsive scaffolds are usually manufactured by modifying or functionalising common scaffold materials. The simplest way to obtain magnetic-responsive scaffolds and integrate MNPs in the three-dimensional structure is to dip-coat the scaffolds into aqueous ferrofluids containing MNPs with several polymers or to disperse them throughout the scaffold. Yet, several studies already reported the direct obtention of magnetic scaffolds by introducing magnetic materials directly into the scaffold production line. In terms of available techniques, the conventional ones such as sacrificial template, freeze dying, and space holder allow for simple and low-cost manufacturing; however, they do not allow for precise control of the scaffold architecture by itself. In contrast, more innovative manufacturing techniques have been introduced, such as electrospinning, 3D printing, and extrusion-based technologies, that allow for the customization of scaffold shape and structure with high reproducibility and reliability. Yet, these are more expensive techniques, requiring specialized manufacturing devices [47,48].

The sacrificial template technique with polymer sponges is a simple and conventional method to obtain scaffolds with random porous structures. In such cases, the sponge is firstly impregnated with the final scaffold material and then destroyed through a heat-treatment or chemically. This results in a porous interconnected scaffold with specific mechanical properties that will depend on the used materials and on the degree of porosity of the initial sponge. The architecture of the obtained scaffolds using this technique can be controlled to some extent, and it is compatible with various materials such as glass, ceramics, metals, and composites. The main problems of this technique are the lack of reproducibility between samples and also that complex shapes are difficult to obtain. This technique was used by Bigham et al. to design a new magnetic nanocomposite, Mg_2_SiO_4_-CoFe_2_O_4_ scaffold. These multifunctional magnetic scaffolds were produced to be used in hyperthermia-based therapy and localized drug delivery. Their characterization showed that they had an interconnected porosity and desirable mechanical properties, close to trabecular bone. The in vitro analysis also showed that their physiochemical and biological properties, such as bioactivity and biodegradability, have potential for bone regeneration [49].

The space holder technique is characterized by the inclusion of porogen particles in the sintered body. The porogen particles are polymers (e.g., polyethylene and starch) or inorganic materials (e.g., sodium chloride and sodium bicarbonate), which are later removed. This technique is cost-effective and provides good control of pore size and mechanical properties by adjusting the size and amount of the porogen particles. For example, Salmani et al. developed a magnetic scaffold by combining several ceramics with MNPs and mixing them with porogen particles of sodium bicarbonate and sodium chloride. In the end, the porogen particles were removed by sintering the compressed material at 850 °C, revealing a final porous structure with magnetic properties [50].

The freeze-drying technique involves sudden and directional freezing to yield porous ceramics; the technique takes advantage of ice crystals to form columnar porous structures instead of inclusion of organic materials. Once the formation of long and oriented ice crystals is carried out, the crystals are sublimated, followed by exposing the scaffold to a high temperature to be consolidated. Due to the oriented structure of the crystals, the mechanical properties of the resulting scaffold are usually satisfactory but also dependent on the used materials, and the process is both energy and time-consuming. Ge et al., produced using this technique Fe_3_O_4_/chitosan magnetic scaffolds, where a suspension of Fe_3_O_4_ MNPs was mixed with an acidified chitosan solution and frozen at −20 °C followed by freeze-drying for 4 days. The obtained scaffold presented a porosity of around 80%, which decreased as the content in MNPs increased. The composite presented magnetic properties and could sustain cell proliferation with no toxicity effects [51].

Among the innovative methods, the first case is electrospinning. This versatile and simple production technique adopts an electric field to obtain fibrous scaffolds in nanometric and micrometric scales. Using this technique, the scaffolds are obtained from both polymer and polymer/ceramic solutions. The obtained scaffolds have great potential, due to a structure of nanofibers mimicking the extracellular matrix [52]. Among their greatest advantages, the electrospun materials offer a high surface area to volume ratio, potential for the release of drugs, controllable fiber diameters, and high porosity and permeability [53]. In terms of production conditions, the flow rate, applied voltage, solution concentration, collector distance, and the solution’s conductivity and volatility are the parameters related to the production of these scaffolds, particularly to manipulate the porosity, the pore size, and the fiber shape. Estévez et al. designed magnetic and biocompatible electrospun scaffolds, combining type-I collagen and SPIONs. Electrospinning specificities included a 22 G tip, 12 cm distance between the tip and the collector, a voltage between 20 and 22 kV and a flow rate between 400 and 300 µL h^−1^. This led to the production of nanostructured scaffolds composed of randomly oriented collagen fibers, where superparamagnetic nanoparticles were embedded. The scaffolds preserved the magnetic properties of the nanoparticles, making these matrices excellent candidates to explore the use of magnetic stimuli for biomedical applications. Furthermore, the biological assessment of these collagen scaffolds confirmed high viability, adhesion, and proliferation of both pre-osteoblastic cells and human bone marrow-derived mesenchymal stem cells [54]. Similarly, Li et al. also produced magnetic scaffolds via electrospinning. In this case, Fe_3_O_4_ MNPs and icariin (ICA) were introduced into polycaprolactone (PCL) fibers to manufacture PCL/Fe_3_O_4_/ICA scaffolds. In this study, it was proven that it is possible to encapsulate several other molecules and materials into the electrospun fibers, allowing for the introduction of multi-functionalities to the material [52].

Recently, three-dimensional printing technology (3D printing) has emerged as a valuable tool to fabricate biomimetic structures with high precision and accuracy [55]. This technique involves many methods that have applicability and compatibility with different types of materials. Generally, printing works based on a programmed 3D image, and it generates a scaffold through the deposition of layers. This usually happens using bio-ink, in the form of hydrogels or viscous fluids [56]. This technique has significant advantages such as reproducibility, precise control on the porosity, pore size and shape, and tunable mechanical properties. Petretta et al. developed multifunctional and resistant 3D structures using 3D printing by combining PCL-based scaffolds with hydroxyapatite and SPIONs. The scaffolds were produced with high reproducibility and shape control. Additionally, the in vitro results showed that the magnetic scaffolds guaranteed a good proliferation and intrinsic osteogenic potential, indicating no toxic effects on cells [57]. Furthermore, De Santis et al. prepared, via 3D printing, fully biodegradable magnetic scaffolds, made using PCL matrix and iron-doped hydroxyapatite magnetic nanoparticles. These scaffolds were designed to create a cellular microenvironment feasible for bone regeneration, through an external SMF, in order to enhance the cell proliferation [58].

Finally, extrusion-based techniques allow to print a large variety of materials, including polymers, ceramics, and composites at a low cost and with good accuracy. In this technique, the materials were processed as a filament or as a paste, loaded into a nozzle or a syringe fixed on a robotic arm, which will execute the path defined by the slicer. The material is dispensed from the nozzle using heat or pressure. For example, Pan et al. incorporated Fe_3_O_4_ MNPs into a poly-l-lactide (PLA) matrix to obtain a magnetic and biodegradable composite. The in vitro evaluation showed no cytotoxic effect on fibroblasts and enhanced osteogenic differentiation capability [59].

### 4.2. Influence of Magnetic Scaffolds on Bone Regeneration

Bioengineered scaffolds have been at the top of tissue engineering research. For bone tissue, 3D porous constructs present optimal features for cell attachment and communication, which in turn translates into a faster proliferation and differentiation. Using these scaffolds in clinical scenarios reduces problems related to autografts and allografts, since they are designed to avoid rejection at the implant site [60]. However, some of the developed scaffolds fail under in vivo scenarios due to their inert activity, meaning that they only serve as a support for cell migration and not as a stimulant for cell differentiation and proliferation. In bone-related studies, calcium phosphate scaffolds have shown to be the most promising, since this material mimics the inorganic matrix of natural bone. Calcium phosphates also provide chemical stimulation through calcium ions to induce biomineralization [61]. Yet, they present weak mechanical strength, which is a drawback for implants that have to withstand load-bearing conditions. Nowadays, polymers (e.g., collagen, PLA, PCL, and chitosan) are being mixed into inorganic scaffolds to strengthen them and increase the mimicry of the natural bone extracellular matrix [62]. Still, these composites are not perfect due to the lack of cell stimulation and other chemical or physical stimuli that need to be employed.

**Figure 3:**
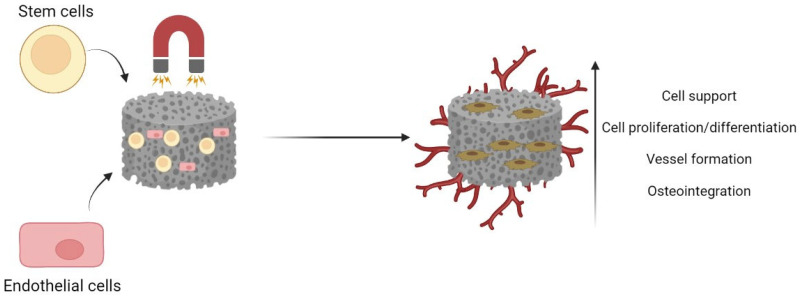
Combination of magnetic bone scaffolds with magnetic fields to promote bone regeneration and angiogenesis.

As shown above, magnetic stimulation is a very promising strategy. Until now, it was summarized that SMFs influence cell behavior, and when combined with magnetic materials such as magnetic nanoparticles, a synergistic effect can be seen in terms of bone tissue regeneration. Since scaffolds are becoming the new norm for bone tissue repair, the scientific community has started to develop magnetic bone scaffolds, mainly by incorporating magnetic particles (micro and nanoparticles) into the matrix of the scaffolds, therefore creating an implantable device that combines the properties of conventional scaffolds, such as cell support, cell migration, cell–cell communication and similarity to the bone extracellular matrix, with the magnetic responsive characteristic of magnetic materials (Figure 3). Thus, Xia et al. showed that a calcium phosphate scaffold loaded with iron oxide nanoparticles was able, in vitro, to sustain stem cell proliferation, and more importantly, the in vivo results showed that by applying an SMF (35 mT), bone formation was improved by 22.2%. This was attributed to the physical forces generated by the magnetic field and to magnetic nanoparticles internalization [63]. Another option is to have scaffolds consisting of electro-spun magnetic polymer fibers. Li et al. included Fe_3_O_4_ nanoparticles inside PCL fibrous scaffolds, which enhanced MC3T3-E1 cell proliferation when the scaffold was combined with an SMF (15 mT) [52]. In another study, with polymeric scaffolds, the authors proved that the introduction of Fe_3_O_4_ nanoparticles, besides introducing magnetic features, also increased the mechanical strength of the scaffold, which is critical in bone regeneration. In fact, by increasing the concentration of nanoparticles, it also resulted in higher cell attachment, since this increases the surface roughness of the material and provides more points of attachment. More importantly, the study reported the synergistic effect of joining the magnetic scaffold to a SMF (1.3 T), where the groups exposed to the magnetic field presented higher mineralization in vitro (up to 50%). The in vivo preliminary results also showed faster bone regeneration [64]. A summary of these studies may be seen in Table 3.

### 4.3. Influence of Magnetic Scaffolds on Angiogenesis

Besides promoting bone cell proliferation for bone growth, angiogenesis must also occur so that sufficient nutrient supply might take place. Although there is a limited number of studies on that topic, some of those report that magnetic scaffolds can also promote blood vessels formation during bone remodeling and repair processes.

As mentioned earlier, co-culture studies between osteoblast and endothelial cells have revealed the interplay between molecules that they secrete, such as BMP2 and VEGF. In an in vitro study by Yun et al., in which a culture of osteoblasts on PCL magnetic scaffolds was externally stimulated by SMF for 3 days, and subsequently treated for HUVECs, tubular cell formation was observed. The combined magnetic stimuli significantly increased the expression of key angiogenic genes, suggesting that the osteoblasts’ heightened functional activity due to magnetism may positively affect endothelial functions [65].

Even though the hydrogel that Wang et al. produced was not used as a bone scaffold, they developed a hydrogel loaded with cobalt ferrite nanoparticles, which under an SMF (80 mT), stimulated early angiogenesis, resulting in faster wound healing [23]. In another study, Filippi et al. developed a hydrogel loaded with Fe_3_O_4_ nanoparticles and adipose-derived cells. After conditioning the scaffold through an SMF of 50 mT, the material was implanted in vivo, and the results showed faster bone regeneration and faster formation of highly vascularized tissues. The authors explained this through mechano-transduction processes that stimulated the VEGF production [66]. A summary of these studies can be seen in Table 3.

## 5. Magnetic Coatings in Bone Tissue Engineering

### 5.1. Production Methods of Magnetic Coatings

Magnetic scaffolds have a beneficial effect on new bone formation, with and without external magnetic stimulation. These findings have paved the way for magnetic coatings. Such coatings may be applied on pre-assembled scaffolds or onto metallic implants used for bone replacement. Despite many advances in the field, metallic implants (especially titanium alloys) remain the gold standard in the clinic due to their price range, biocompatibility, durability, and mechanical properties suitable for load-bearing sites [67,68]. However, they are bioinert, meaning that they do not integrate with the surrounding tissue. This is commonly overcome by coating the implants (the bone-contacting zones) with calcium phosphates, not only preventing the direct contact between bone and metal, that can cause inflammatory reactions leading to fibrous capsule formation and bone loss due to stress shielding, but allowing new bone to be formed at the interface, growing toward the coating, which is a critical factor for implant stabilization [69].

There are several methods of applying a magnetic coating onto a material. Physical and chemical vapor deposition (PVD and CVD) are extensively used to coat orthopedic metallic implants; however, these techniques are not mentioned in the literature regarding magnetic coatings, since they rely on high temperatures for the volatilization of the coating compounds (mostly metals and synthetic high-performance polymers). On the other hand, simple methods that do not require much equipment, such as dip coating, spin coating, and tape casting, have been recurrently mentioned in the scientific literature over the last five to ten years [68,69,70,71,72,73]. Spin coating and tape casting are used in laboratory for magnetic coating application, since these methods are effective for coating flat substrates or substrates with micrometric topography; however, they are not applicable in metallic implants or macrometric 3D scaffold surfaces.

Dip coating is a simple and cheap coating technique that can be performed both manually and automatically, being suitable for small (laboratory) and large (industry) scale, as well as for flat or three-dimensional structures. As the name suggests, the coating is applied by immersing the sample in a solution with the coating molecules. The viscosity, concentration, and temperature of the solution, the immersion duration, and the velocity used to remove the sample from the solution (or the solution from the sample) are the determining factors for the coating thickness. In tape casting, the coating solution is applied directly on top of the substrate, where it dries before being subjected to a final annealing step. In spin coating, the coating solution is also applied on top of the substrate, being evenly distributed due to the rapid rotation of the sample holder [74].

The method we came across the most was electrochemical deposition (ECD) [75,76,77]. In ECD, two electrodes are placed in a solution under an electrical potential difference between electrodes with the coating particles in suspension, and these are directed towards the electrode of opposite charge, containing the substrate material, through an applied electric field that can be generated with direct (DC) or alternated (AC) current [74]. This method, like most, has been adapted in several ways to target specific needs. By changing the applied potential, it is possible to obtain coatings with distinct properties. For instance, it was shown that mineralized collagen coatings deposited by AC-ECD had a higher organic/inorganic mass ratio, as well as a better effect on osteoblast proliferation, than the ones achieved with DC-ECD [78]. The placement of a permanent magnet parallel to the plane of the substrate (during both deposition and drying) also gives rise to a collagen coating with aligned nanofibers [79].

A different method that uses an electrolyte solution and a current discharge to produce magnetic coatings is micro-arc oxidation (MAO), also called plasma electrolytic oxidation (PEO) [80]. It is a fast and affordable process extensively used to form oxide-ceramic coatings on metal alloys to protect them from corrosion. However, this method presents some limitations, as it cannot be used with polymers, only for ceramic coatings, and the final product is often porous and prone to delamination; therefore, additional steps are needed to achieve a durable coating [67,81]. These methods lead to the formation of uniform coatings when dealing with polymers and composites. However, when the goal is to coat using solely ceramics, it often results in poor adhesion to the substrate and cracking of the coating layer, especially if the sintering step is skipped [74]. This drawback can be suppressed by pre-coating the material with polydopamine (PDA) or by adding PDA to the coating solution, eliminating the need for an extra step. PDA can be used to functionalize both organic and inorganic materials. Moreover, it is a human neurotransmitter; hence, it does not have biocompatibility issues, unlike other binding molecules. PDA polymerization will occur if the solution is alkaline, acting as a surface modification on the material that will promote the adhesion of the coating molecules. This system has been reported for iron-oxide magnetic coatings (mainly with the deep-coating technique) [67,68].

### 5.2. Influence of Magnetic Coatings on Bone Regeneration

The presence of magnetic particles on the material has been said to promote osteogenic differentiation of mesenchymal stem cells and adipose-derived mesenchymal cells (ASC), which can be enhanced by the presence of an external magnetic stimulus [77,79,82].

Alexandra Paun, I. et al. spin-coated a microtopographic substrate with 4 wt% MNPs embedded in a mixture of collagen, chitosan, and hydroxyapatite. The coated substrates were seeded with MG63 cells and stimulated for 30 days with three intensities of SMFs (110, 180, and 250 mT). ALP and osteocalcin were systematically upregulated in the samples subjected to the magnetic field, meaning that it promoted differentiation as well as new bone formation. Alizarin Red staining also revealed that stimulated samples exhibited higher mineral content, which is in accordance with the two previous results. All these results were directly proportional to the intensity of the magnetic field, meaning that magnetic fields up to 250 mT, applied on cells seeded onto magnetic coatings, accelerated new bone formation [71]. The only parameter that was found to be downregulated in this study was cell proliferation (in this case, the higher the intensity of the magnetic field, the lower the proliferation). Other studies have reported this, and it is believed that the SMF provokes a reduction of the proliferation due to the early differentiation occurrence [71,73].

Besides the direct effects of magnetism on cell behavior, it is also reported that a certain deformation can occur in polymeric materials containing magnetic nanoparticles when stimulated by an external magnetic field. This deformation is thought to act as mechanical stimulus on the surrounding cells [83]. Concerning this aspect, Zhuang, J. et al., coated a titanium (Ti) surface with mineralized collagen containing magnetite nanoparticles using AC-ECD. The application of an SMF (100 mT) caused the coating layer to expand and contract, giving rise to an internal mechanical stimulus, sensed by the cells surrounding the implant. Hence, mechanical stimuli triggered the upregulation of osteoblast-differentiation-related genes and the activity of a series of proteins that ultimately resulted in enhanced differentiation of MC3T3-E1 cells [75]. As a follow-up to this work, the same coating was tested with MSCs under periodic mechanical stimulus, and the results obtained were similar to those regarding SMF [76].

The same group studied the different properties of magnetic collagen coatings with randomly organized and aligned collagen fibers (MCC and A-MCC) [77,79]. These were produced by AC-ECD, and for the A-MCC, a magnet was placed in parallel to the substrate during the coating deposition and drying. The relative composition and the saturated magnetization of the two coatings did not show significant differences; nonetheless, cellular responses were not similar. BMSCs align with the collagen fibers of the A-MMC and present a more elongated morphology when compared to the cells seeded on the MMC. The first study did not use an external magnetic field, since the goal was to compare solely the two magnetic coatings; however, they concluded that the aligned coating resulted in enhanced cell proliferation and differentiation with upregulation of several osteogenic markers [79].

In a later study, the cellular assays were carried out under the influence of SMF (280 mT) in several directions in order to understand if stimuli direction had any differential effects on cellular behavior. The A-MCC coatings were tested with SMFs parallel to the coating collagen fibers, while the A-MCC-F were tested parallel to the substrate but perpendicular to the aligned collagen fibers, ꞱA-MCC-F, and perpendicular to the coating, ꞱA-MCC [77]. The cells subjected to a magnetic field in the same direction of the collagen fibers in the coating were also aligned in the same direction, and their differentiation markers were upregulated while the two perpendicular stimuli caused a downregulation of the differentiation of BMSCs, as well as disorganized cellular distribution [77].

To this day, only a few in vivo studies have been carried out on magnetic coatings. Lin, S. et al. tested the previously described MCC on full-thickness bone defects created on rat skulls, with and without an SMF of 300 mT, placed in parallel with the implanted structure to further evaluate the response caused by the directed stimuli. After 8 weeks, the newly formed bone on the SMF-stimulated animals was denser than that found on the non-stimulated animals. Moreover, angiogenesis appeared to be enhanced in the first case as well [77]. The group proved that the magnetic coating and magnetic field had synergic effects in promoting osteogenesis in vivo. However, only the randomly organized magnetic coating was tested and only under parallel magnetic stimulation; therefore, the differential effect of stimulus direction in vivo cannot be extrapolated [77].

The previously described iron oxide/PDA coating was also tested in vivo. The coated Ti scaffolds were implanted onto femoral critical-size defects in rabbits, and their osteointegration with and without magnetic field influence was evaluated. Both micro-CT scanning and histological staining revealed that the magnetic coating enhanced new bone formation in the scaffold when compared with the controls (Ti scaffold and Ti scaffolds coated with PDA). As expected, new bone deposition was increased due to the presence of the external SMF [68]. A summary of these studies can be seen in Table 4.

## 6. Further Discussion

Stimulation is known to be a key parameter in any tissue regeneration strategy. In bone, either natural healing or biomaterial-assisted healing depend on chemical, physical, or a combination of both for an optimal and faster regeneration after injury. As mentioned above, chemical stimulation through ions (e.g., calcium, phosphate, iron, magnesium) or small molecules such as growth factors present several limitations. Among them, the low availability of ions or the instability and price of growth factors make these strategies difficult to implement in terms of production and translation into clinics. On the other hand, physical stimuli such as electric or magnetic fields are being studied as a more feasible alternative. In fact, special attention must be taken regarding the great potential of magnetic fields, as their easy production and safety for the human body make them very attractive when compared to electrical fields where safety is a major concern.

Since most biological tissues where bone is also included present diamagnetic properties, it is natural that they respond to the influence of an external magnetic field. To date, the influence of magnetic fields on bone cells has been described as a positive reinforcement in terms of cell proliferation, and differentiation in the case of stem cells. The biological mechanism is related to several factors such as protein expression and increased ionic uptake. Magnetic fields have been associated with the activation of membrane channels that increase the uptake of calcium and iron ions; the ions then increase the production of ROS that will further induce proliferation and differentiation. Even though magnetic fields’ influence on humans has been studied for decades, there are still many gaps in the literature in terms of cell response. Most studies report the fundamental markers of cell proliferation (e.g., cellular metabolic activity) and differentiation (e.g., ALP activity); however, no studies go deeper, especially in vasculature-associated cells. Specific analyses such as genomics, lipidomics, proteomics, and metabolomics will provide a general knowledge on how different cells respond to magnetic fields of different strengths.

In terms of magnetic-responsive materials, MNPs have been extensively used for all kinds of applications, both in biomedicine and in other fields of science. The general advantages of MNPs are very well-described and are recognized among experts in the scientific community. In terms of regeneration, MNPs already proved that they can induce bone regeneration by promoting cell proliferation and differentiation. Yet, some studies also report that the MNPs are inert unless a magnetic field is applied. This discrepancy can be attributed to the differences between nanoparticles, even if the main material is the same. As mentioned before, iron oxide is the most common material among MNPs. However, a slight change in the synthesis method can influence the final product. For example, a simple change in the atmospheric conditions of synthesis can produce slightly more oxidized particles, which in turn can significantly influence the saturation magnetization and consequently affect how the material responds to a magnetic field [84]. Capping agents, size, and morphology may also play essential roles in the cells’ response [28]. This proves that much more fundamental research must be done, mainly focusing on normalizing these studies in order to obtain a more accurate and reliable answer as to how cells respond to MNPs with and without a magnetic field.

Finally, in terms of bone tissue engineering, several studies report that the combination of magnetic scaffolds or coatings with SMFs bring beneficial responses in terms of bone regeneration and implant integration. In general, the great majority of studies indicate that the combination of magnetic scaffolds with SMFs increase cells proliferation and differentiation, due to increased nanoparticle internalization and as a result of the mechanical forces that the fields exert on the magnetic material. However, it was found that this research is more focused on the translation side, since the studies show that cells proliferate in these materials, a phenomenon enhanced by the stimulation with SMF, but rarely study the cellular mechanisms involved in the process. Along with an increasing percentage of elderly population, musculoskeletal diseases tend to also increase, and translational research is crucial in order to develop functional materials; however, fundamental research could also improve the already developed materials and provide clues that would speed up the process such as better material selection, correct external field application, and better structural scaffold design.

Another topic of discussion is the lack of studies regarding angiogenesis. As mentioned earlier, angiogenesis is critical in bone remodeling and should occur simultaneously with bone regeneration in order to supply oxygen, nutrients, and growth factors. From the small amount of available literature on this topic, it seems that SMF can also modulate endothelial cell growth, with the production of VEGF and mechano-transduction processes as the currently available mechanisms. Still, in tumoral situations, MNPs were found to inhibit angiogenesis through ROS formation, which serves as a therapy for cancer but also shows a toxic profile for regeneration. Yet, tumoral conditions are very different from bone injuries, namely, the acidic pH (~6.0) of the tumoral microenvironment and the higher concentration of H_2_O_2_. At such pH, iron-containing nanoparticles can catalytically convert the excess of H_2_O_2_ into ROS and in this way damage cells [85]. In the case of bone regeneration, pH and H_2_O_2_ levels are normal, and in this scenario, MNPs may not be involved in ROS production. Still, caution should be taken regarding endothelial cell cytotoxicity, since these cells are known for being more sensitive to oxidative stress.

## 7. Conclusions

This review shows that SMFs have great potential in aiding bone tissue regeneration. This external stimulus induces cell proliferation and differentiation by activating receptors that modulate the uptake of vital ions in cell modulation. Moreover, protein activation and cell cytoskeleton alterations through SMFs are also normal responses that promote bone regeneration. Due to the easy production of SFM and the easy manipulation of their strength, much can be adapted in order to achieve a proper cell response with a large range from a few mT to tens of T.

In terms of magnetic responsive materials, scaffolds that incorporate MNPs work as multifunctional materials that promote the correct support for bone and endothelial cell growth and proliferation. Their porous structure allows for cell communication and expansion throughout the scaffold, which in turn makes possible an optimal osteointegration. Additionally, by combining SMF, an enhanced cell proliferation and differentiation are observed, proving that SMF can act as a physical stimulus that can replace the common chemical one, serving as a safer, cheaper, more stable, and readily available aid in bone healing. The same was observed in coatings for metallic implants, where the synergistic strategy improved implant osteointegration.

In conclusion, magnetic bone tissue engineering has shown sufficient results as a viable option for promoting bone regeneration, and due to the increased number of bone fractures, more clinical studies should be carried out in order to create medical devices. Yet, the involved cellular mechanisms in bone regeneration through magnetic fields are still poorly understood, and for this reason, more fundamental research work should be performed, such as in metabolomics, proteomics, and genomics.

## Figures and Tables

**Table 1:** Summary of studies reporting the influence of magnetic fields on bone regeneration and angiogenesis.

Type of Magnetic Field			Type of Cell	Results	Results In Vivo	Mechanism	Reference
Static	Pulsed	Intensity					
X	__	500 nT 0.2 T 16 T	MC3T3-E1	Concentration of minerals increase alongside the strength of SMF; higher degree of differentiation	_____	Osteoblasts in differentiation accumulated more mineral elements than non-differentiated cell cultures	[18]
X	__	16T	MC3T3-E1	Iron accumulation inside the cells	_____	Expression of transferrin receptor one and ferroportin 1; associated with increased ALP activity and mineralization	[14]
X	__	4 mT	___	_____	Prevent bone deterioration in diabetic mice	Treatment led to high levels of osteocalcin; increased number of osteoblasts; upregulation of *BMP2* and *Runx2* genes	[21]
X	__	60 uT 120 uT	HUVECs	40% increase in proliferation	_____	Endothelial cells’ functionality increased with the application of SMF, which upregulated eNOS expression	[22]
__	X	___	___	Increase in endothelial cells in the metaphysis of long bones	Prevent bone loss in a mouse model of postmenopausal osteoporosis	PEMF-induced osteogenesis and expansion of types H vessels may be mediated by HIF-1α signaling in these endothelial cells	[23]

**Table 2:** Summary of studies reporting the influence of magnetic nanoparticles on bone regeneration and angiogenesis.

Material	Methods	Type of Magnetic Field			Type of Cell	Results	Results In Vivo	Mechanism	Reference
		Static	Other	Intensity					
Iron oxide nanoparticles	Coprecipitation	X	___	0.3–0.45 T	Stem cells	Temporary decrease in cell proliferation and viability	_____	Iron in magnetized MSCs aggravated the loss of viability	[42]
Fe_3_O_4_/BSA nanoparticles (200 nm)	Desolvation	X	___	1 T	Stem cells	Increase in the differentiation of stem cells into osteogenic cells	_____	Higher level of nanoparticle internalization; proliferation decreased, probably due to cell differentiation	[43]
Co_0.5_Mn_0.5_Fe_2_O_4_@PMMA	Microwave-driven non-hydrolytic approach	X	___	0.2 T	Pre-existing bone cells	Restore the balance of osteoblasts and osteoclasts activity in the condition of osteoporosis	_____	Stimulated integrins, improving preosteoblast activity and inhibiting osteoclasts	[44]
Gelatinous sponges with SPIONs	Coprecipitation	__	X	1.5 T 7 T	___	_____	Increased bone density and trabecular volume; new bone formation and blood vessel formation in the sockets of rats	SPIONs are taken up by osteoblasts and vascular endothelial cells, leading to improved bone formation and blood vessel formation	[45]
PEI-coated SPIONs	Coprecipitation	__	__	___	HUVECs	Negatively affected the functionality of primary HUVECs; decreased blood vessel number at tumor sites	_____	SPIONs lead the cells to produce more reactive oxygen species, disrupting the cells’ actin cytoskeleton activity	[46]

**Table 3:** Summary of studies reporting the influence of magnetic scaffolds on bone regeneration and angiogenesis.

Material	Methods	Type of Magnetic Field			Type of Cell	Results	Results In Vivo	Mechanism	Reference
		Static	Other	Intensity					
Calcium phosphate scaffold with IONPs	Compression	X	__	35 mT	hDPSCs	Formation of bone enhanced by 22.2%	_____	Probably due to physical forces generated by the magnetic field and the presence of magnetic nanoparticles within the scaffold	[63]
PCL fiber scaffold with IONPs	Depressurization of subcritical CO_2_ fluid	X	___	15 mT	MC3T3-E1	Cell growth	_____	Great cell penetration, collagen deposition and angiogenesis	[52]
Polymeric scaffold with IONPs	Two-photon polymerization	X	___	1.3 T	MG-63	Increased mineralization up to 50%	Faster bone regeneration in initial animal tests	SMFs promote cell attachment and the early-stage mineralization of nanoparticle-free osteoblast-like cells	[64]
PCL-MNP scaffold	Space holder	X	___	15 mT	HUVECs	Tubular cell formation	_____	Increase expression of key angiogenic genes	[65]

**Table 4:** Summary of studies reporting the influence of magnetic coatings on bone regeneration.

Material	Methods	Type of Magnetic Field			Type of Cell	Results	Results In Vivo	Mechanism	Reference
		Static	Other	Intensity					
Chit/Col matrix with Hap and IONPs	Spin coating	X	__	110 mT 180 mT 250 mT	MG-63	Enhanced cell differentiation and tissue mineralization Reduced cell proliferation	_____	Probably due to deformations of the structure caused by the SMF, providing strain stimulation to the cells	[71]
Collagen with IONPs	Electron capture dissociation (AP-ECD)	X	___	100 mT	MC3T3-E1	Enhanced osteogenic differentiation	_____	Deformation of the coating under SMF provides mechanical stimulation to the cells	[75]
IONPs and Polydopamine (PDA)	Electron capture dissociation (AP-ECD)	X	___	15 mT	hBMSCs	Enhanced proliferation and osteogenic differentiation	Increased mineralization and new bone formation	Upregulation of osteogenic factors and TGFβ-Smads signaling pathway	[76]
Collagen with IONPs	Electrodeposition	___	X	1500 and 2800 Oe	MSCs	Enhanced cell adhesion, proliferation, and differentiation	_____	Upregulation of integrin β1 and of YAP/TAZ transcription factors	[79]
Nickel nanowires	Electrodeposition	___	X	4 mT	ASCs	Osteogenic conversion of the ASC	_____	Topography of the coating and MF caused tensile and shear forces on the cell membrane	[82]
Collagen with IONPs	Eletrochemical deposition	X	___	280 and 300 mT (in vitro and in vivo, respectively)	BMSCs	Upregulation of differentiation markers	Increased bone formation Enhanced angiogenesis	Direction of the SMF parallel to the collagen alignment in the coating promoted directional mechanical stimulation.	[77]

## Abbreviations

ASC	Adipose-Derived Mesenchymal Cells
ALP	Alkaline Phosphatase
BSA	Bovine Serum Albumin
CVD	Chemical Vapor Deposition
DPSCs	Dental Pulp Stem Cells
ECD	Electrochemical Deposition
eNOS	Endothelial Nitric Oxide Synthase
bFGF	Fibroblast Growth Factor
ICA	Icariin
MNPs	Magnetic Nanoparticles
MAO	Micro Arc Oxidation
PEO	Plasma Electrolytic Oxidation
PVD	Physical Vapor Deposition
PCL	Polycaprolactone
PDA	Polydopamine
PEG	Polyethylene Glycol
PEI	Polyethylenimine
PLA	Poly-l-lactide
PMMA	Polymethyl Methacrylate
ROS	Reactive Oxygen Species
SMF	Static Magnetic Field
SPIONS	Superparamagnetic Iron Oxide Nanoparticles
VEGF	Vascular Endothelial Growth Factor
